# Teaching and training for general practice: a Dutch academic success story

**DOI:** 10.3399/bjgpopen17X100917

**Published:** 2017-05-03

**Authors:** Harm WJ van Marwijk

**Affiliations:** 1 GP & Clinical Chair in General Practice Research, Centre for Primary Care, Division of Population Health, Health Services Research and Primary Care, Manchester, UK

**Keywords:** general practice, teaching, training

An image of a dead canary in a carbon monoxide filled coal mine springs to mind when I think of the crisis affecting the GP workforce and NHS. As an experienced GP, researcher, and teacher from the Netherlands, I looked forward in 2015, to start working in the strongest academic primary care environment in the world. These last 2 years, the opportunities for GP research have indeed been better than in the Netherlands, but my overall impression is that, unfortunately, general practice itself is at the brink of collapse. I am not sure how my research would add to its sustainability. In this article, I briefly describe under what conditions GPs in the Netherlands successfully countered such crumbling forces and why academic structures in the Netherlands may have been more successful in supporting GPs.

## Conditions

The first condition for good primary care and its teaching and training is a protective societal environment. To write that physicians in the UK do not perceive their government to be protective of primary care is probably an understatement. On the other hand, the Dutch government seems more supportive. For example, it cooperates with the Dutch College of General Practitioners (DCGP), universities, and other national organisations of GPs in what is called a ‘polder’ model: all working towards a consensus. GPs are highly organised and are seen as equal partners in care, although scaling and substitution of hospital care are also regarded as eroding the GPs’ direct contact with the patient. It would seem that Dutch GPs have overall bargained better for their professional autonomy.

The second condition pertains to the specifics of the Dutch healthcare system compared to the NHS. The Dutch Healthcare Reform Act of 2006 introduced health insurance companies while keeping universal healthcare coverage. GPs are still free at the point of care. These companies now cover care risk and allow customers a baseline care package at a basic insurance premium. If people want to pay more for more care, they cover more. A key element is annual risk equalisation between companies.^1^ The changes have led to more bureaucracy for GPs, but may have strengthened the GPs’ overall bargaining position and increased their overall sense of ownership of care. Dutch GPs still provide 24-hour care. As is to be expected, primary care may be more expensive in the Netherlands.^2^

The third condition relates to the DCGP's long-term emphasis on helping GPs to deal with uncertainty, by co-creating GP-relevant knowledge. That has led to good GP–specialist training programmes that are well integrated into academic departments of general practice. These programmes focus on self-directed learning on the job, on interpersonal and consultation skills,^3,4^ and on learning to use evidence that is translated into GP-specific guidelines or standards.

## Why GP standards are a good idea 

Many standards are symptom- and not disorder-focused. The DCGP considered that multidisciplinary guidelines, such as the National Institute for Health and Care Excellence guidelines, have little use to GPs, as they offer no specific contextual guidance. GPs define their own levels of tolerance of uncertainty, and communicate those widely. While the standards policy has been instrumental in helping Dutch GPs define their work and fight turf battles with specialists, GPs in the UK seem to be in a weaker position, as they lack such specific guidance. A paternalistic secondary care attitude is not helpful for the type of systems-wide collaboration the NHS now needs to help it deal with the older population with long-term conditions (LTCs) and multimorbidity. A partnership of equals is the only working model, acknowledging uncertainty and different perspectives.^5^

The standards have also given Dutch specialists a much clearer idea of what to expect of GPs, and many have come to realise that they can do little without GPs, but when collaborating with them, they can achieve more. To let specialist colleagues in the UK get away with a disregard for the interdependence between settings is probably not a good idea. Students and young GPs are responding to a general lack of respect towards primary care, and as canaries, are flying away to other countries. We have a responsibility to define together what everybody's work is in health care.

Perhaps there are lessons to learn in the UK. There is an alarming 40% shortage of GPs. Among the 60% that are still working, GP burnout is rife and urgently requires structural changes to engage doctors again.^6^ Instead, more team members are introduced into larger practices that have a defensive culture and shun direct communication and accountability. Relationship building, open conversations, and personal continuity give way to abstract procedures and commercially-focused digital solutions. There seems an unusually strong disconnect between teaching, research, and practice here in the UK. GPs need to start redefining the content of their work themselves again; otherwise their jobs end up like the miners around Manchester.
